# Purification, structure and anti-oxidation of polysaccharides from the fruit of *Nitraria tangutorum* Bobr.

**DOI:** 10.1039/c8ra01125g

**Published:** 2018-03-27

**Authors:** Baotang Zhao, Jing Liu, Xin Chen, Ji Zhang, Junlong Wang

**Affiliations:** College of Food Science and Engineering, Gansu Agricultural University Lanzhou 730070 China zhaobaotang@126.com +86-0931-7631201; Bioactive Products Engineering Research Center for Gansu Distinctive Plants, Northwest Normal University Lanzhou 730070 China

## Abstract

In this paper, polysaccharides were extracted from the fruits of *Nitraria tangutorum* Bobr. (NTWP) using a hot water extraction method and extraction conditions were optimized by RSM. The optimal conditions were determined as follows: extraction time 7 h, extraction temperature 60 °C, ratio of water to raw material 15 : 1, and with these conditions, the yield was 14.01 ± 0.11%. After purification using DEAE-cellulose column and Sephadex G-200 column, NTWP-II was successfully obtained. The results of GC-MS and SEC-LLS analysis suggested that monosaccharide composition of NTWP-II was composed of Rha, Ara, Man, Glc and Gal with the molar ratio of 1.14 : 2.5 : 3.00 : 2.69 : 5.28 and *M*_w_, *M*_w_/*M*_n_ and *R*_z_ 2.29 × 10^5^, 1.32, 15.22. The detailed structure of NTWP-II was characterized by FT-IR, NMR. Based on these analyses, the structure of the repeating unit of NTWP-II was established.

## Abstract 2

Antioxidant activity of NTWP-II, evaluated *in vitro*, indicates that NTWP-II has good potential as a natural antioxidant used in the food industry.

## Graphical abstract

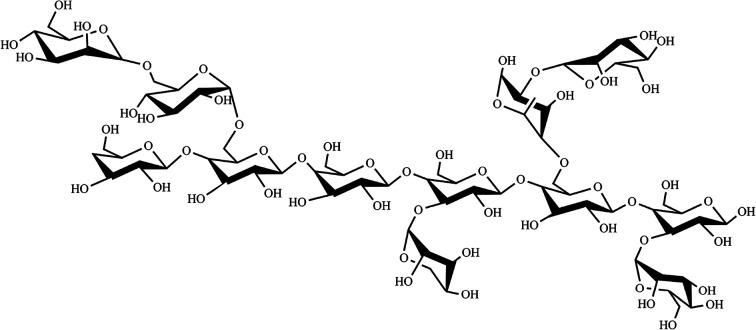

## 1. Introduction

Oxidation is an important energy production process in organisms. However, it is well-known that free radicals, especially reactive oxygen species such as hydrogen peroxide, hydroxyl radicals and superoxide which generate during metabolism, could damage fatty acids, proteins, DNA and other macromolecules in our bodies and result in various diseases including cardiovascular diseases, neurodegenerative diseases, cancers and aging-related disorders. Recent papers prove that synthetic antioxidants are potential hazards in liver damage, carcinogenesis *et al.* and are restricted.^1,2^ Thus, it is very important to exploit natural antioxidants, especially those of plant origins. Constituents and crude numerous plant extracts have been recognized as natural antioxidants, with beneficial effects against free radicals in biological systems.

*Nitraria* (Zygophyllaceae) includes 15 species and only *Nitraria tangutorum* Bobr. grows in China.^3^ It is used to sand stabilization because of natural ability in withstanding wind and sand.^4^ In addition, the fruits and seeds of *Nitraria tangutorum* Bobr. were often used by local residents to treat ailments of the spleen and stomach, indigestion, neurasthenia and colds, and the leaves were used as an antispasmodic, antineuropathic, and antiarrhythmic agent in folk medicine.

Thus, there was a growing interest in the area of research on the positive effect of *Nitraria tangutorum* Bobr. on human health and other benefits. Recent studies focused on ingredients such as seed oil, anthocyanins, tangutorine, polysaccharides *et al.* from their leaves, seeds, fruits and juice by-products.^2–8^ Some researches indicated that seed oil has been shown to improve immune response, reduce oxidants, and mitigate fatigue.^4–6^ Nine anthocyanins were separated from the seed oil of *Nitraria tangutorum* by subcritical fluid extraction.^5^ Cyanidin derivatives, the main components, has a good antioxidant.^4^ François' research showed that hydroalcoholic extract from the fruits of *Nitraria sibirica* Pall. induced vasorelaxation.^9^ From the leaves of *Nitraria tangutorum* Bobr., Duan and Liu isolated tangutorine (1) and identified its structure.^6,7^ The fruits of *Nitraria tangutorum* Bobr. were traditional medicinal food of Tibetans and used to alleviate fatigue caused by oxygen deficiency for thousands of years. Wei hua Ni' study indicated that water-soluble polysaccharides from the fruits of *Nitraria tangutorum* Bobr. in Tibetan plateau significantly exhibited anti-fatigue activities for the first time, through triglyceride mobilization during exercise and protecting corpuscular membrane by prevention of lipid oxidation *via* modifying several enzyme activities.^8^ Zhang *et al.* reported that the polysaccharides from *Nitraria* fruit was composed of mannose, rhamnose, galacturonic acid, glucose, galactose and arabinose with approximate molar ratios of 9.2 : 3.3 : 1.1 : 1 : 1.9 : 2.3.^10^ Ni *et al.* isolated the polysaccharides (NTWP) from *Nitraria tangutorum* Bobr. by hot water extraction, purified by DEAE-cellulose ion exchange chromatography and found that NTWP was composed of mannose, rhamnose, glucuronicacid, galacturonic acid, glucose, galactose and arabinose with approximate molar ratios of 3.9 : 1.8 : 0.2 : 3.3 : 70.6 : 7.6 : 13.1.^8^ Although previous studies have reported the structural properties of the polysaccharides from the fruits of *Nitraria*, the results of these studies were not consistent. To scan the structural characterization of polysaccharides from *Nitraria tangutorum* Bobr., we purified NTWP by DEAE-cellulose anion-exchange chromatography and Sephadex G-200 column, identified their structural features by GC-MS, SEC-LLS, FT-IR and NMR. Furthermore, we evaluated antioxidant activity of NTWP-II *in vitro*.

## 2. Materials and methods

### 2.1. Plant materials

The fruits of *Nitraria tangutorum* Bobr. were collected from Min qin in Gansu, China at October 2012. The fresh fruits were air-dried, crushed and kept in plastic bags at room temperature for use.

### 2.2. Chemicals and reagents

Pyridine, acetic anhydride, 1,1-diphenyl-2-picrylhydrazyl (DPPH), butyl hydroxy anisd (BHA), nitro blue tetrazolium (NBT), dihydronicotineamidadenine dinucleotide (NADH), phenazinemethosulfate (PMS), ethylene diamine tetraacetic acid (EDTA), trifluoroacetic acid (TFA) and thiobarbituric acid (TBA) were purchased from Sigma Aldrich (St. Louis, MO, USA), and DEAE-cellulose 52 was purchased from the Pharmacia Co. (Upp-sala, Sweden). The solvents for size exclusion chromatography coupled with laser light scattering (SEC-LLS) and gas chromatography-mass spectrometry (GC-MS) were of chromatographic purification. All other reagents were of analytical grade.

### 2.3. Preparation of NTWP

The extraction process was carried out by the former report with slight modification.^8^ The dried fruit powder was defatted with petroleum ether, and refluxed with petroleum benzine to remove lipids, some colored ingredients and small molecular impurities. After removing the solvent, the resulting pretreated powder was dried and used for the extraction of NTWP. The pretreated samples were soaked in distilled water at room temperature. For hot water treatment, a sample (1 g) was put into a triangular flask (1 L), diluted with distilled water at a liquid–solid ratio varying from 8 : 1 to 25 : 1, v/w, and extracted at 30–80 °C for 1–12 h. The mixture was centrifuged at 5000 rpm for 15 min, and the insoluble residue was retreated as mentioned above for 2 times. The supernatants were collected, concentrated by a rotary evaporator under reduced pressure to an appropriate volume, then the supernatant was deproteinized by using Sevage's method. Then the deproteinized supernatant was dialyzed, concentrated, mixed with four times of absolute ethanol and kept overnight at 4 °C. The resulting precipitates were collected by centrifugation at 5000 rpm for 10 min, washed sequentially with anhydrous ethanol and acetone, and dried to afford crude NTWP. The extraction yield (%) was calculated with the formula below:1
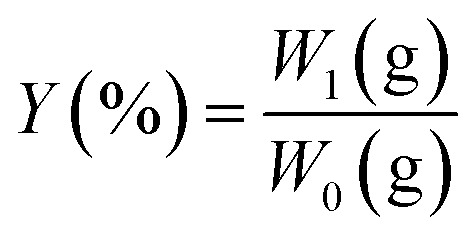
where *Y* was the yield of polysaccharide (%), *W*_1_ was the polysaccharides of extraction (g), and *W*_0_ represented dried sample weight (g).

### 2.4. Experimental design of NTWP

Based on the results of single factor experiments, a three-level BBD with three factors was applied to determine the optimal levels for extraction temperature (*X*_1_), extraction time (*X*_2_) and ratio of water to raw material (*X*_3_), which significantly affected the extraction efficiency, and the optimal range of each variable was determined. The experimental designs of the code and the actual levels of each factor are presented in Table 1. As shown in Table 1, the three factors chosen for this study were designated as *X*_1_, *X*_2_ and *X*_3_ and were prescribed into three levels, coded +1, 0, and −1 for high, intermediate and low value, respectively.

**Table 1:** Independent variables and their levels used in the response surface design^a^

Independent variables	Symbol		Factor level		
	Coded	Uncoded	−1	0	1
Extraction time (h)	*x*_1_	*X*_1_	5	7	9
Extraction temperature (°C)	*x*_2_	*X*_2_	50	60	70
Ratio of raw material to water (mL mg^−1^)	*x*_3_	*X*_3_	15 : 1	20 : 1	25 : 1

a *x*_1_ = (*X*_1_ − 7)/2; *x*_2_ = (*X*_2_ − 60)/10; *x*_3_ = (*X*_3_ − 20)/5.

Test variables were coded according to the following equation:2
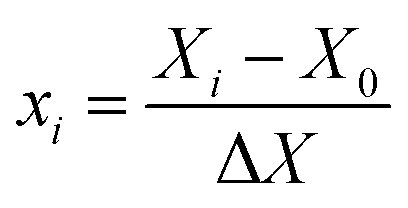
where *x*_*i*_ was the coded value of an independent variable; *X*_*i*_ was the actual value of an independent variable; *X*_0_ was the actual value of an independent variable at centre point; Δ*X* was the step change value of an independent variable. The whole design consisted of 17 experimental points carried out in random order (Table 2).

**Table 2:** Experimental results of the response surface methodology

No.	*X*_1_ extraction 4time (h)	*X*_2_: extraction temperature (°C)	*X*_3_: ratio of water to material (mL g^−1^)	Y: extraction yield^a^ (%)
1	−1	−1	0	12.015
2	1	−1	0	12.09
3	−1	1	0	12.405
4	1	1	0	12.75
5	−1	0	−1	11.46
6	1	0	−1	10.885
7	−1	0	1	11.745
8	1	0	1	12.21
9	0	−1	−1	9.885
10	0	1	−1	12.51
11	0	−1	1	12.91
12	0	1	1	10.805
13	0	0	0	14.19
14	0	0	0	14.04
15	0	0	0	14.475
16	0	0	0	13.95
17	0	0	0	13.875

a Mean of triplicate determination.

All experiments were performed in triplicate and the averages of polysaccharide yields were taken as response. For predicting the optimal point, a second order polynomial model was fitted to correlate relationship between independent variables and response (polysaccharide yield). For the three factors, the equation was:

3 *Y* = *A*_0_ + ∑*A*_*ix*_*i* + ∑*A*_*ii*_*X*_*i*2_ + ∑*A*_*ij*_*X*_*i*_*X*_*j*_

where *Y* was the response variables (yields of polysaccharides in real values). *A*_0_, *A*_*i*_, *A*_*ii*_, *A*_*ij*_ were the regression coefficients of variables for intercept, linear, quadratic and interaction terms, respectively. *X*_*i*_ and *X*_*j*_ were independent variables (*i* ≠ *j*).

The significance in the model was evaluated by analysis of variance (ANOVA). The accuracy and general ability of the polynomial model could be evaluated by a determination coefficient *R*^2^ and adjusted coefficient of determination *R*_adj_^2^. Subsequently, several confirmation experiments were conducted to verify the validity of the statistical experimental strategies. The regression coefficients were then used to make statistical calculation to generate dimensional and contour maps from the regression models. Statistica (Version 8.0, USA) software package was used to analyze the experimental data. *P*-values of less than 0.05 were considered to be statistically significant.

### 2.5. Isolation and purification of NTWP

The crude polysaccharide (NTWP), obtained under the optimal conditions, was redissolved in distilled water, and the supernatant was loaded onto a DEAE-cellulose column (2.6 cm × 50 cm), eluting stepwise with a gradient of aqueous solution of sodium chloride (NaCl, 0–1.0 M) at a flow rate of 0.2 mL min^−1^. The eluate was collected by using an automated fraction collector. In order to detect polysaccharides, a 0.2 mL sample collected from each eluted fraction (2 mL per tube) was mixed with sulfuric acid and phenol to produce color reaction. The carbohydrate content of each tube was monitored at 490 nm. The fractions with rose color were combined, concentrated, dialyzed, and lyophilized, after which the main polysaccharide was redissolved in deionized water and loaded onto a Sephadex G-200 column (20 × 500 mm). After loading the sample onto the column, the column was eluted with deionized water at a flow rate of 0.5 mL min^−1^. The main polysaccharide fractions were collected, combined, and lyophilized to obtain a purified polysaccharide (NTWP-II).

### 2.6. Structural characterization of NTWP-II

#### 2.6.1. Components analysis

The carbohydrate and proteins contents of NTWP-II were determined by the phenol-sulphuric acid method and Bradford's method according to the method by our report. The composition was analyzed according to the earlier report from our laboratory.^11^ Briefly, 4 mg of sample were dissolved in 4 mL of 4 M trifluoroacetic acid acetate (TFA) in a test tube and then hydrolysed at 120 °C for 10 h under air-tight conditions. TFA was then evaporated through decompression and distillation. When the tube was dry, 10 mg of ammonium hydrochloride and 0.5 mg pyridine were added and allowed to react in a 90 °C water bath for 30 min. Then 0.5 mL of cold (kept at 4 °C in a refrigerator) acetic anhydride was added to the test tube and the mixture was incubated in the 90 °C water bath for a further 30 min to allow the acetylation reaction to occur. The end-product was decompressed and distilled to dryness. The acetate derivatives were analysed by GC-MS with an HP-5 capillary column (HP 6820, Hewlett-Packard). The temperature programme was set to increase from 120 °C to 250 °C with an increment of 5 °C min^−1^ and He was the carrier gas. The standard monosaccharides were measured following the same procedure. d-Amr, l-Rha, d-Lyx, d-Ara, d-Xyl, d-Man, d-Glu, d-Gal were used as references.

#### 2.6.2. FT-IR analysis

The sample was ground with KBr powder and then pressed into pellets for Nicolet NEXUS 670 FT-IR in the frequency range of 4000–400 cm^−1^ to detect functional groups. Sixteen scans at a resolution of 4 cm^−1^ were averaged and referenced against air.

#### 2.6.3. Molecular weight determination

Size exclusion chromatography, combined with laser light scattering (SEC-LLS), measurements were carried out with a multi-angle laser photometer (MALLS, *k* = 690 nm; DAWN EOS, WyattTechnology Co., USA). An UltrahydrogelTM column (7.8 × 300 mm, Waters, USA) was used as the SEC instrument. An Optilab refractometer (Dawn, Wyatt Technology Co., USA) was simultaneously connected. The polysaccharide solutions with desired concentrations were prepared, and optical clarification of the solutions was achieved by filtration into a scattering cell. The injection volume was 200 μL, and the flow rate was 0.5 mL min^−1^. The refractive index increment (d*n*/d*c*) value of the sample was determined, by using an Optilab refractometer at 690 nm and 25 °C, to be 0.147 mL g^−1^. The basic light scattering equation is as follows:4
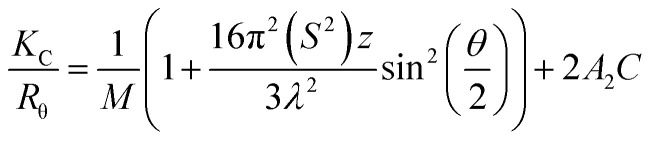
where, *K* is an optical constant equal to [4π^2^*n*^2^(d*n*/d*c*)]/(*λ*^4^*N*_A_), *c* is the polysaccharide concentration in mg mL^−1^, *R*_θ_ is the Rayleigh ratio, *λ* is the wavelength, *n* is the refractive index of the solvent, d*n*/d*c* is the refractive index increment, *N*_A_, the Avogadro number, *N*_A_ is the second virial coefficient. As the column separates the polymer according to molecular weight, each fraction was led to the light scattering detector for instantaneous measurement of the scattering intensities. The refractive index detector, connected in series, gave the estimate of the polymer concentration. In chromatography mode, we have a single and sufficiently low concentration at a particular slice because of the further dilution by the SEC column of the already dilute injected solutions.

#### 2.6.4. NMR spectroscopy

NMR spectra were recorded with a BRUKER400 MHz spectrometer at a probe temperature of 80 °C. All exchangeable H of the samples were replaced by D_2_O before obtaining the spectra in deuterium oxide. Chemical shifts were calibrated using coaxial NMR tubes with (3-trimethylsilyl)-propane sulfonic acid sodium salt (DSS, dH = 0.00, dC = 0.00) in the inner tube. The 2D ^1^H-^1^H correlated spectroscopy (COSY), ^1^H-^1^H total correlation spectroscopy (TOCSY), ^1^H-^13^C heteronuclear single quantum coherence (HMQC) and ^1^H-^13^C heteronuclear multiple quantum coherence (HMBC) measurements were used to assign signals and to determine the sequence of sugar residues.^12^

### 2.7. Assay for antioxidant activities

Polysaccharides were dissolved in deionized water at the concentration of 0.02–5 mg mL^−1^. Antioxidant assay *in vitro* was carried out on scavenging DPPH, hydroxyl, superoxide radical and chelating metal, using V_E_, V_C_, BHT and EDTA as the positive controls according to the earlier report.^11^ Each concentration was measured in triplicate and averaged.

## 3. Results and discussion

### 3.1. Influence of three single factors on the extraction efficiency of NTWP

#### 3.1.1. Effect of ratio of water to raw material on extraction yield of NTWP

**Fig. 1:**
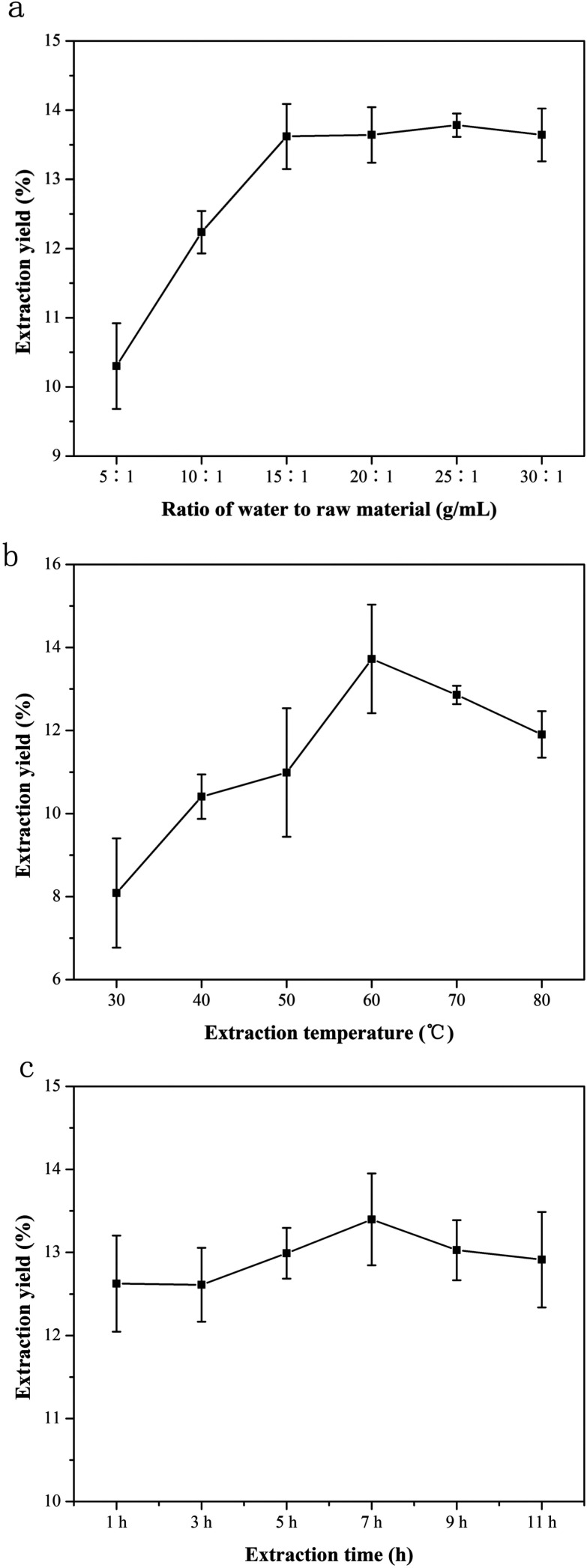
Effect of ratio of water to raw material, extraction time and extraction temperature ((a) extraction temperature 55 °C, extraction time 5 h; (b) extraction time 5 h, ratio of water to raw material 40 : 1 (v/w); (c) ratio of water to raw material 40 : 1 (v/w), extraction temperature 55 °C). Data are shown as mean ± SD (*n* = 3).

Ratio of water to raw material was a routine parameter that significantly affects the extraction efficiency. In the present study, extraction of NTWP was carried out at different ratios of water to raw material (5, 10, 15, 20, 25, 30 mL g^−1^) when other parameters were as follows: extraction temperature 55 °C and extraction time 5 h. As shown in Fig. 1a, the extraction yield increased with the increase of ratio of water to raw material from 5 to 15 mL g^−1^ and while above 15 mL g^−1^, the yield increased slowly up to its maximum amount of 13.42 ± 0.4% at 20 mL g^−1^ when ratio of water to raw material continued to rise. This phenomenon could be explained that the higher the ratio of water to raw material was, the lower the concentration and viscosity of the extraction solvent would be. Hence more polysaccharides molecules could dissolve in water and the extraction yield increased.^13^ But the amount of polysaccharides in raw material was definite and further increase of ratio of water to material would not increase the extraction yield. Thus, ratio of water to raw material range of 15 : 1 to 25 : 1 was favorable for extracting NTWP.

#### 3.1.2. Effect of extraction temperature on extraction yield of NTWP

Extraction time is another factor that influenced the extraction efficiency. To investigate the effect of temperature on extraction yield of NTWP, extraction was conducted at different temperatures (30, 40, 50, 60, 70 and 80 °C, respectively), when the other extraction variables were set as follows: ratio of water to raw material 20 mL g^−1^ and extraction time 5 h. The effect of extraction time on the yield is presented in Fig. 1b. As shown in Fig. 1b, the extraction yield increased when temperature increased from 30 to 60 °C, reaching a maximum (13.30 ± 0.6%) at 60 °C, and then declined when extraction temperature continued to rise. Therefore, it could be explained that as the temperature increased, the polysaccharides diffusion coefficient would increase, resulting in an enhanced solubility of the polysaccharides in the solvent. However, when temperature became higher, the extraction yield of NTWP decreased drastically, probably due to that high temperature could destroy the structure of polysaccharides and lead to degradation. Therefore, extraction temperature range of 50–70 °C was favorable for extraction of NTWP, being selected for further optimization in BBD design.

#### 3.1.3. Effect of extraction time on extraction yield of NTWP

To investigate the influence of time on extraction efficiency, extraction process was carried out at 1, 3, 5, 7, 9, 11 h, while other parameters were as follows: ratio of water to raw material 20 mL g^−1^ and extraction temperature 60 °C. The effect of extraction time on the yield was presented in Fig. 1c. When extraction time varied from 1 to 9 h, the variance of extraction yield was relatively rapid and reached the maximum extraction yield (13.72 ± 1.3%) at 7 h, and then decreased as the extraction proceeded. These results can be explained that extended extraction time (>7 h) would result in the degradation of the polysaccharides, which was induced by their thermal instability. Therefore, after the maximum extraction yield was achieved, longer time of how water extraction was not necessary. Thus, temperature range of 5–7 h was selected as optimal in the BBD experiment.

### 3.2. Optimization of extraction conditions of NTWP by BBD

#### 3.2.1. Predicted model and statistical analysis

As shown in Table 1, each experiment in the design matrix was performed and the experimental data were obtained. By applying multiple regression analysis on the experimental data, the Design-Expert software generated a second order polynomial equation that could express the relationship between process variables and the response. The final equation obtained in terms of coded factors was given below:

5 *Y* = 14.11 + 0.039*X*_1_ + 0.2*X*_2_ + 0.37*X*_3_ + 0.067*X*_1_*X*_2_ + 0.26*X*_1_*X*_3_ − 1.18*X*_2_*X*_3_ − 0.87*X*_1_^2^ − 0.92*X*_2_^2^ − 1.66*X*_3_^2^

where *Y* was the predicted yield of polysaccharides, *X*_1_ was ratio of water to raw material (W), *X*_2_ was extraction temperature (°C) and *X*_3_ was extraction time (h).

Analysis of variance (ANOVA) was performed to evaluate the predictive model and the variables. The *P*-values was used as a tool to check the significance of each coefficient and indicated the pattern of interactions between variables. The smaller the value of *P* was, the more significant the corresponding coefficient would be. A significant lack of fit demonstrated that the fitted model failed to failed to represent the data in the experimental domain at which points were not included in the regression. The ANOVA for the fitted quadratic polynomial model of extraction efficiency was presented in Table 3. As shown in Table 3, the quadratic regression model has a very low *P*-value (*P* < 0.0001), indicating that the fitness of the model was highly significant. The “Model *F*-Value” of 69.92 implied the model was significant. There was only a 0.01% (*P* < 0.0001) chance that a “Model *F*-Value” could occur due to noise. At the same time, the “Lack of Fit *F*-Value” of 0.48 implied the lack of fit was not significant relative to the pure error. A “Lack of Fit *F*-Value” this large has 71.48% (*P* = 0.7148) chance to occur because of noise. Coefficient of determination (*R*^2^) indicated that 98.90% of the variations can be explained by the fitted model. The value of adjusted determination coefficient (*R*_adj_^2^) was 0.9749, which also confirmed that the model was highly significant. Moreover, a relatively low value of coefficient variation (1.68%) indicated high degree of precision and good deal of reliability for the experimental values. “Adeq Precision” measured the signal to noise ratio. A ratio greater than 4 was desirable. The ratio of 26.961 indicated an adequate signal. Thus, the results indicated that this model could be used to navigate the design space.

**Table 3:** Analysis of variance of the experimental results of the BBD

Variables	Sum of squares	DF	Mean square	*F* value	*P* value
Model	27.51	9	3.06	69.92	<0.0001
*X*_1_	0.012	1	0.012	0.27	0.6163
*X*_2_	0.31	1	0.31	7.05	0.0327
*X*_3_	1.07	1	1.07	24.55	0.0016
*X*_1_ *X*_2_	0.018	1	0.018	0.42	0.5391
*X*_1_ *X*_3_	0.27	1	0.27	6.19	0.0418
*X*_2_ *X*_3_	5.59	1	5.59	127.95	<0.0001
*X*_1_^2^	3.20	1	3.20	73.20	<0.0001
*X*_2_^2^	3.56	1	3.56	81.39	<0.0001
*X*_3_^2^	11.59	1	11.59	265.17	<0.0001
Residual	0.31	7	0.044		
Lack of fit	0.081	3	0.027	0.48	0.7148
Pure error	0.23	4	0.056		

The significance of each coefficient was also determined by using *F*-value and *P*-value. It could be seen that the linear coefficients (*X*_2_ and *X*_3_), quadratic terms coefficients (*X*_1_^2^, *X*_2_^2^, and *X*_3_^2^) and cross product coefficients (*X*_1_*X*_3_, *X*_2_*X*_3_) were significant, with small *P*-values (*P* < 0.05). And the significant interaction between factors means that effect of them on the extraction yield of polysaccharides was not simply an additive. The empirical model was converted to three-dimensional (3D) and contour plots to predict the relationships between the independent variables and the response.^14,15^

#### 3.2.2. Optimization of extraction procedure

**Fig. 2:**
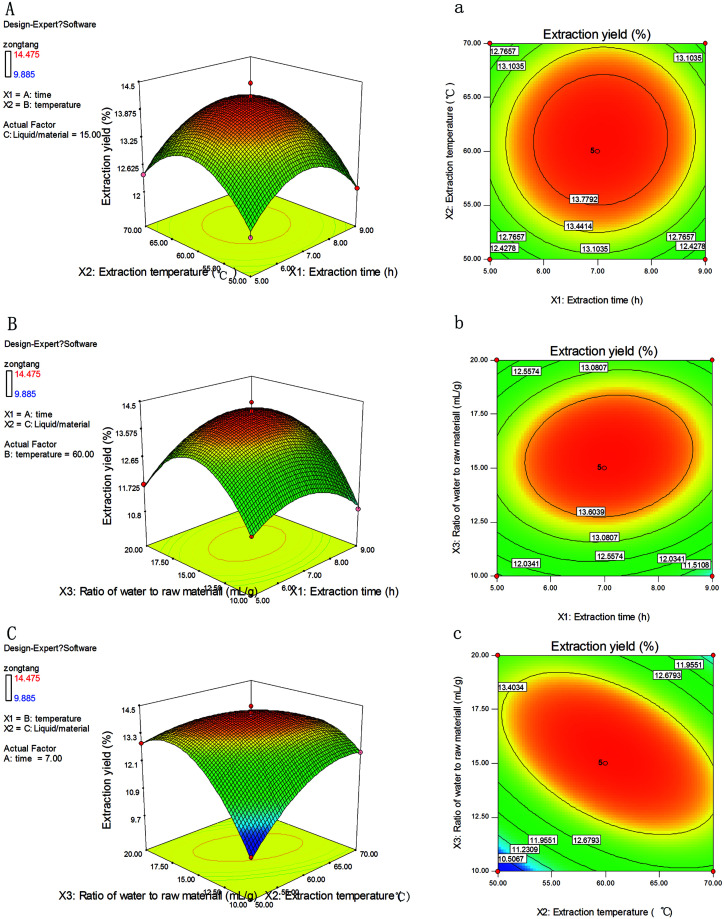
Tri-dimensional response surface and contour plots showing the experimental factors and their mutual interactions on NTWP-II extraction ((a) extraction time and extraction temperature, (b) extraction time and ratio of water to raw material, (c) extraction temperature and ratio of water to raw material).

The graphical representation of regression equation was obtained using Design-Expert to evaluate the effects of independent variables and their interactions on extraction efficiency of NTWP. As graphical representations of the regression equation, three dimensional response surface and two dimensional contour plots were very useful to visualize the relationship between independent and dependent variables and the interactions between two variables. Different shapes of the contour plots indicated different interactions between the variables. Circular contour plot means the interactions between the corresponding variables are negligible, while elliptical contour suggested the interactions between the corresponding variables are significant. Among these three variables (ratio of water to raw material, extraction temperature and extraction time) as shown in Fig. 2, one variable was kept constant at zero level, when the other two variables within the experimental range were depicted in the plots.

The extraction yield (*Y*) of NTWP affected by extraction time (*X*_1_) and extraction temperature (*X*_2_) was shown in Fig. 2A and a with ratio of water to raw material (*X*_3_) fixed at a zero level. The extraction yield (*Y*) increases rapidly when ratio of extraction time (*X*_1_) and extraction temperature (*X*_2_) increase in the range of 5–6.8 h and 50–60 °C, respectively; but beyond 6.8 h and 60 °C, extraction yield (*Y*) decreases slightly. The elliptical contour plot shown in Fig. 2A, a indicated the mutual interactions between extraction time (*X*_1_) and extraction temperature (*X*_2_) were significant.

Fig. 2B, b showed the 3D response surface plot and the 2D contour plot at varying extraction time and ratio of water to raw material at fixed extraction temperature (0 level). The same trends with Fig. 2A, a were depicted in Fig. 2B, b, of which Fig. 2B, b showed a similar elliptical contour plot. The extraction yield of polysaccharides affected by extraction temperature and ratio of water to raw material is shown in Fig. 2C, c with extraction time fixed at a zero level (7 h). The yield of polysaccharides increased evidently with the extraction temperature from 50 °C to 65 °C. However, beyond 65 °C, the extraction yield would not increase as the temperature ascended.

#### 3.2.3. Verification of predictive model

By employing the software Design-Expert, the solved optimum values of the tested variables were extraction time 7.08 h, extraction temperature 60.45 °C, liquid–solid ratio 15 : 49. Under the optimal conditions, the maximum predicted yield of NTWP was 14.13%. Taking account of the operating convenience, the optimal parameters were determined as following: extraction time 7 h, extraction temperature 60 °C, liquid–solid ratio 15 : 1.

To ensure the predicted result was not biased toward the practical value, experimental rechecking was performed using this deduced optimal condition. A mean value of 14.01 ± 0.11% (*n* = 3), obtained from real experiments, demonstrated the validation of the RSM model. The good correlation between experimental and predicted values confirmed that the response model was accurate and adequate for the extraction of NTWP. The validation result revealed that there was no significant difference between experimental and predicted values, suggesting that the response model was adequate for reflecting the expected optimization.

### 3.3. Isolation and purification of polysaccharide fraction NTWP

**Fig. 3:**
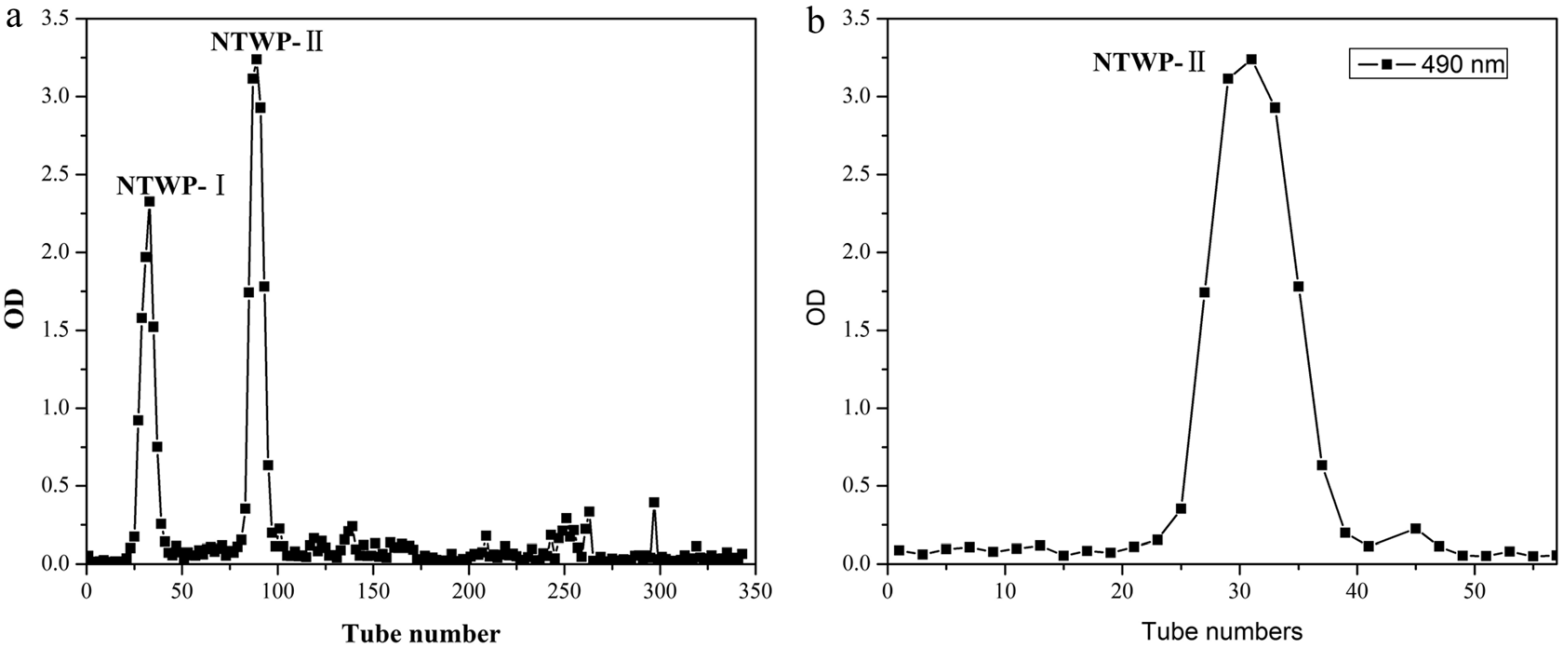
Elution curve of NTWP on DEAE-cellulose column (a) and elution curves of NTWP-II on Sephadex G-200 column (b).

NTWP solution (5 mg mL^−1^) was loaded into a DEAE cellulose-52 column equilibrated with a linear gradient elution of NaCl from 0 M to 1.0 M. Two independent elution peaks (NTWP-I 24.11% and NTWP-II 64.57%, Fig. 3A) detected at 490 nm by the phenol–sulfuric acid assay were obtained.

NTWP-I consisted of glucose and NTWP-II consisted of rhamnose, mannose, glucose, galactose and arabinose by GC-MS. The next research focused on NTWP-II. Thus, only the NTWP-II fraction was collected for the subsequent purification and antioxidant activity assays. The NTWP-II fraction was collected, dialyzed, concentrated, and loaded into a Sephadex G-200 column. The fraction produced a single elution peak (Fig. 3B).

### 3.4. Chemical composition of NTWP-II

**Fig. 4:**
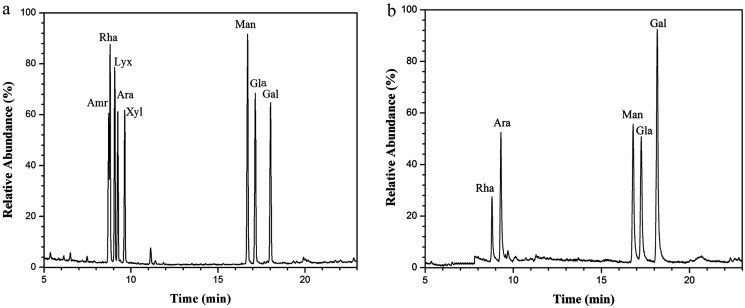
The GC-MS chromatograms of derivatives of 8 standard monosaccharides (a) and component monosaccharides released from NTWP-II (b). Peaks: (1) d-Amr, (2) l-Rha, (3) d-Lyx, (4) d-Ara, (5) d-Xyl, (6) d-Man, (7) d-Gla, (8) d-Gal.

NTWP-II isolated from the fruit of *Nitraria tangutorum* Bobr. by a series of purification procedures, including water extraction, ethanol sedimentation, deproteinization, and dialysis. The total sugar content of NTWP-II separated by Sephadex G-200 column was 94.3 ± 3.42% and was poor in protein (2.22 ± 0.34%). Through acid hydrolysis, NTWP-II was subjected to GC-MS analysis and the results were shown in Fig. 4. As can be seen in Fig. 4a, NTWP-II was composed of rhamnose, arabinose, mannose, glucose and galactose with the molar ratio of 1.14 : 2.5 : 3.00 : 2.69 : 5.28. These results didn't show a good correlation with those reported by Ni *et al.* They found that NTWP was composed of mannose, rhamnose, glucuronicacid, galacturonic acid, glucose, galactose and arabinose with approximate molar ratios of 3.9 : 1.8 : 0.2 : 3.3 : 70.6 : 7.6 : 13.1. This result might be due to regional difference.

### 3.5. Molecular weight determination

**Fig. 5:**
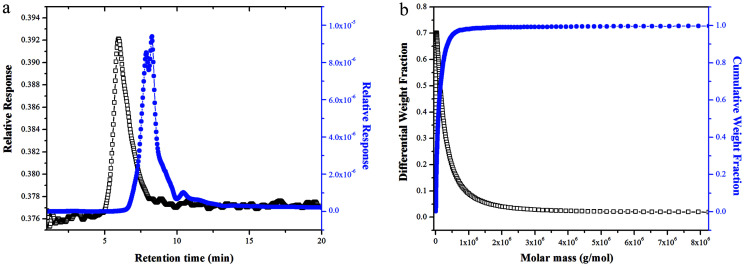
SEC-LLS chromatograms of the samples.((a) Laser light scattering photometry and RI for NTWP-II and (b) molar mass distribution analysis of NTWP-II).

The SEC-LLS chromatogram patterns of NTWP-II were shown in Fig. 5a and b. The chromatograms of NTWP-II exhibited a single peak indicating that there was no aggregation and the homogeneity of the purified samples. The molecular weight of NTWP-II was determined by SEC-LLS. The weight average molar mass (*M*_w_), polydispersity (PD, *M*_w_/*M*_n_) and *z*-average radius of gyration (*R*_z_) were 2.29 × 10^5^, 1.32, 15.22, respectively.

### 3.6. FT-IR analysis of NTWP-II

**Fig. 6:**
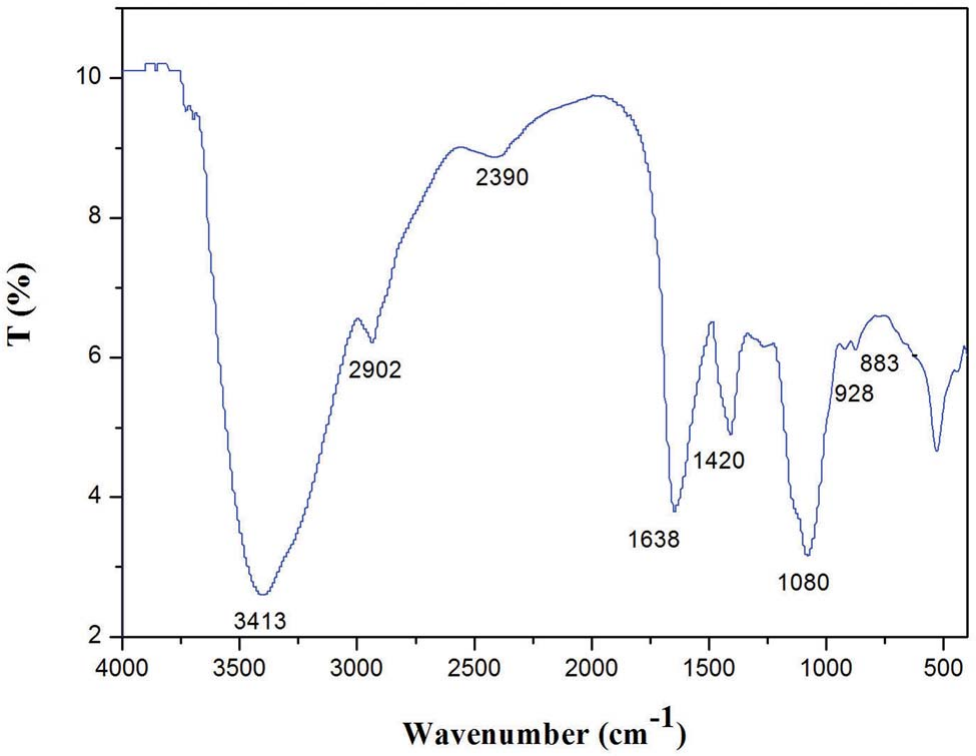
FT-IR spectroscopy of NTWP-II.

NTWP-II was characterized by FT-IR spectroscopy as shown in Fig. 6. The infrared spectra showed strong and wide stretching peak around 3413 cm^−1^ for O–H stretching vibrations as well as a weak absorption peak at 2902 cm^−1^for C–H stretching vibrations. A strong absorption peak at 1618 cm^−1^ was attributed to asymmetric and symmetric stretching of the carboxylate anion group (COO), indicating NTWP-II be acidic polysaccharides. Each particular polysaccharide has a specific band in the 1000–1200 cm^−1^ region. This region was dominated by ring vibrations overlapped with stretching vibrations of (C–OH) side groups and the (C–O–C) glycosidic band vibration. The absorptions at 1072.99 cm^−1^ indicated a pyranose form of sugar.^16,17^ A specific band in the 1080 cm^−1^ region indicated a pyranose form of sugar.^17,18^ In the anomeric region (950–700 cm^−1^), the spectrum exhibited the characteristic absorption at 883 cm^−1^ due to the presence of mannose.^12^ This showed a good correlation with monosaccharide composition. These results indicated that NTWP-II possessed typical absorption peak of polysaccharides.

### 3.7. Nuclear magnetic resonance (NMR) spectroscopy analysis

**Fig. 7:**
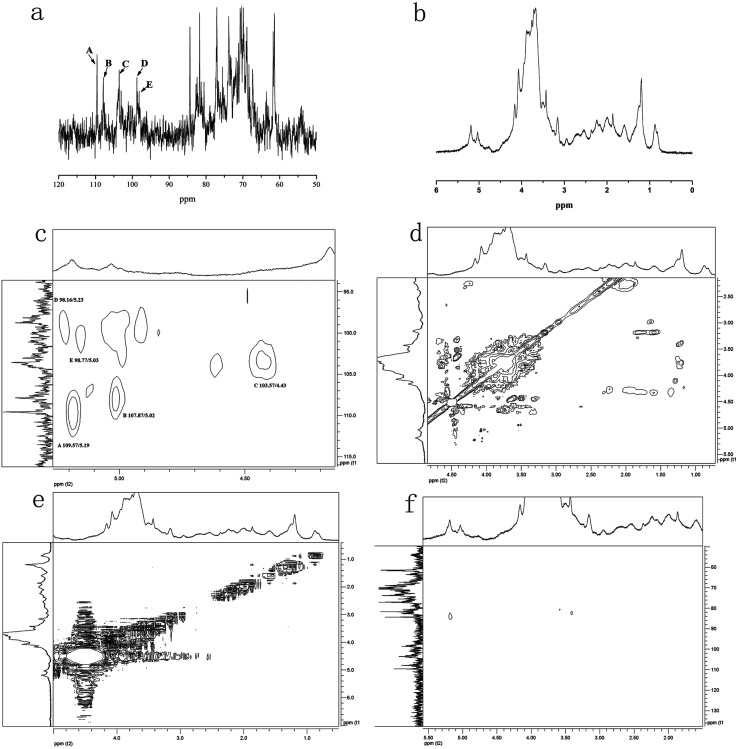
NMR spectra of the sulfated polysaccharide NTWP-II. (a) ^1^H NMR spectrum; (b) ^13^C NMR spectrum; (c) ^1^H-^13^C HMQC spectrum; (d) ^1^H-^1^H TOCSY spectrum; (e) ^1^H-^13^C HMBC spectrum; and (f) ^1^H-^1^H NOESY.

NMR spectroscopy could provide detailed structural information including the monosaccharide composition, α- or β-anomeric configurations, linkage patterns, and sequences of the sugar units. Signals of NTWP-II in 1D ^1^H and ^13^C NMR and 2D NMR (HMQC, HMBC, COSY, NOESY and TOCSY) spectra were assigned as completely as possible, based on the monosaccharide analysis and chemical shifts reported in the literature.^20,21^ The ^1^H and ^13^C NMR spectra of NTWP-II were shown in Fig. 7a and b. The ^1^H NMR spectrum was crowded in a narrow region ranging from 3 to 5 ppm which was typical of polysaccharides, and this confirmed the presence of many similar sugar residues.^22,23^

^1^H NMR spectrum (Fig. 7b) NTWP-II contained five signals at *δ* 5.19, 5.02, 4.43, 5.23 and 5.03 ppm for the anomeric protons, indicating for five residues which were designated as A, B, C, D and E, respectively, present in NTWP-II. The anomeric protons of residues A (5.19 ppm), B (5.02 ppm), D (5.23 ppm) and E (5.03 ppm) has the chemical shifts larger than 5.0 ppm and very small *J*-coupling constants of ^3^*J*_H-1,H-2_ (the values of ^3^*J*_H-1,H-2_ was 2.17, 2.23, 0.86 and 3.84 Hz, respectively), suggesting that residues A, B, D and E were α-linked. Meanwhile the anomeric proton of residue C has the chemical shift smaller than 5.0 ppm and suggested residue C being β-linked.^20,21,24^ The 1D ^13^C NMR spectra (Fig. 7a) showed five signals in the anomeric region ranging from *δ* 90 to *δ* 110. These results were consistent with the fact that the NTWP-II consisted of five types of monosaccharides. Furthermore, the cross-peaks of *δ* 109.57/5.19, 107.87/5.02, 103.57/4.43, 98.16/5.23, and 98.77/5.03 ppm were observed in 2D HMQC NMR spectrum (Fig. 7c), indicating that the anomeric carbon signals at *δ* 109.57, 107.87, 103.57, 98.16 and 98.77 ppm corresponded to the anomeric proton signals at *δ* 5.19, 5.02, 4.43, 5.23 and 5.03, respectively.

In TOCSY and HMQC spectrum (Fig. 7d and c), *δ* 61.44 ppm and *δ* 68.35 ppm were attributed to no-substitution of C-6 and weak substitution of C-6. Based on the chemical shifts of H, C and ^3^*J*_H-1,H-2_ of residues C, residues C was identified as β-galactose. According to the molar ratio of monosaccharide, signal intensity of ^13^C spectrum (C-1 *δ* 103.57 ppm and C-3 *δ* 75.50 ppm) and weak substitution of C-6, it was concluded that residue C was (1→3)-β-galactose and the main sugar units of backbone. The anomeric carbon signals at *δ* 109.57 of residues A indicated that the position of the glycosidic linkage of A was C-1. Based on standard monosaccharide and former research, residues A was identified as α-arabinose, because the chemical shifts of C-1 (*δ* 97.6 ppm) in α-arabinose was larger than in β-arabinose (*δ* 93.4 ppm). The chemical shifts of C-4 (*δ* 71.19 ppm) down low field confirmed the substitution at position C-3. Therefore, we inferred that residue A was (1→4)-α-arabinose. Compared with standard monosaccharide, residues E was identified as (1→)-α-mannose, residues D and B were identified as (1→)-α-glucose and (→6)-α-rhamnose, respectively from the chemical shifts of residues D (C-6 *δ* 63.53 ppm) and B (C-1 *δ* 107.87 ppm).

In NOESY spectrum (Fig. 7f), the crosspeaks between C H-3 and E H-1, A H-1 and C H-3 indicated that the presence of the bond (1→3) between C and E, A and C. In HMBC spectrum (Fig. 7e), some crosspeaks were observed between H-1 and a ^13^C peak in different residues: E C-1 and B H-2, and E C-1 and D H-6. These HMBC data revealed the existence of two sequences in the structure: E (1→2) B, E (1→6) D.
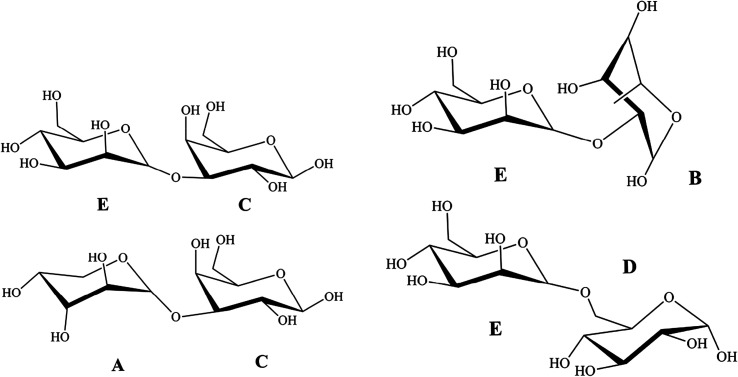


According to previous studies of NTP-II, (1→3)-β-galactose was the main sugar units of backbone and linked with α-mannose, α-arabinose and β-galactose by the glycosidic linkage of (1→3) and (→1)-α-glucose of the side chain was obtained. It was proposed that the residue sequence in the repeating unit was as:
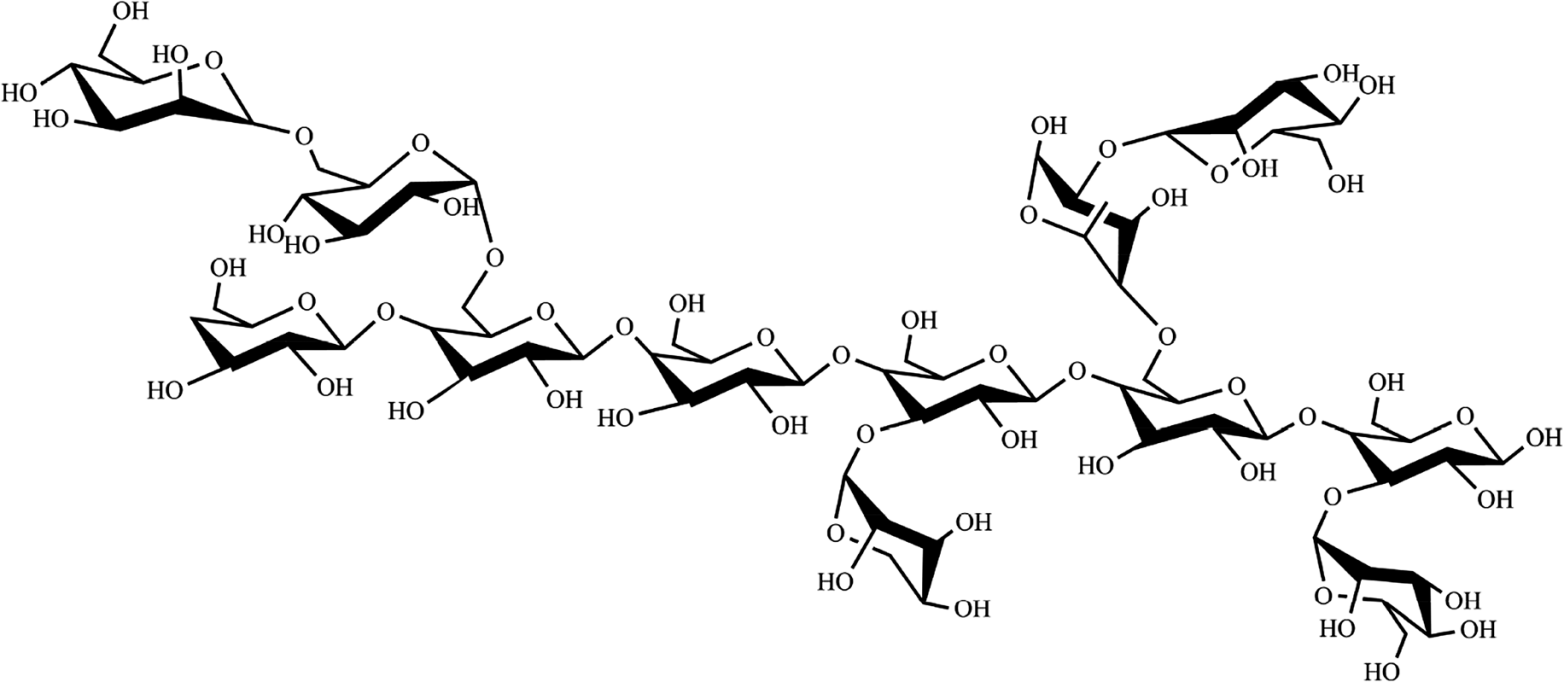


### 3.8. Antioxidant activities *in vitro* of NTP-II

**Fig. 8:**
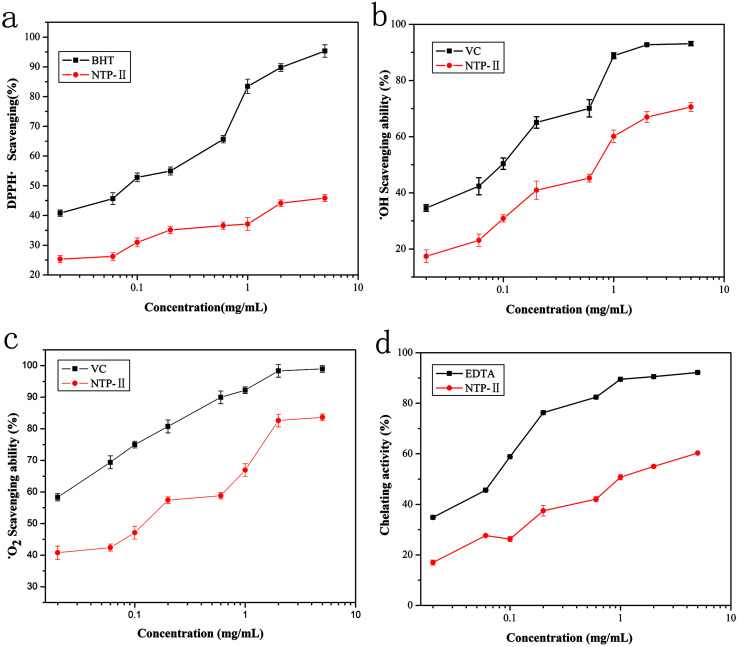
Antioxidant activity of NTWP-II at different concentrations ((a) scavenging activity of NTWP-II and BHT at different concentrations on DPPH radicals, (b) scavenging activity of NTWP-II_E_ and V_C_ at different concentrations on hydroxyl radicals, (c) scavenging effects of NTWP-II and V_C_ at different concentrations on superoxide radical, (d) chelating activity of NTWP-II and EDTA at different concentrations on ferrous ion). Data are shown as mean ± SD (*n* = 3).

DPPH radical was a stable free radical, of which alcohol solution has characteristic absorption maximum at 517 nm and used to evaluate the free radical scavenging activities of natural compounds. When DPPH ethanol solution was reduced, absorbance was decreased and the solution changed from purple to light yellow.^17^ The reduction extent of absorbance reflects the hydrogen or electron donating abilities of antioxidants.^25^ As shown in Fig. 8a, at the range from 0.02 mg mL^−1^ to 1 mg mL^−1^, the color significantly became light with the increase of NTWP-II concentrations. Beyond the concentration of 1 mg mL^−1^, the trend was not significant. However, the scavenging effect of NTWP-II on DPPH radical was lower than that of BHT. Previous studies demonstrated that the antioxidant activity of polysaccharides has been attributed to their composition and structural features.^26^ The results implied that NTWP-II could have stronger ability to donate electron or hydrogen.^25^

Hydroxyl radical could cause severe damage to adjacent biomolecules or cell death.^19^ For hydroxyl radical, there were two types of antioxidation mechanism: one suppresses the generation of the hydroxyl radical, and the other scavenges the hydroxyl radicals generated. Thus, it forms a stable radical to terminate the radical chain. It has been found that the antioxidant activities of polysaccharides are affected by various factors such as chemical contents, molecular mass and structure.^27^ The scavenging effect on hydroxyl radical of NTWP-II was shown in Fig. 8b. The hydroxyl radical, known to be generated through the Fenton reaction in this system, was scavenged by samples. For NTWP-II, the effects of scavenging hydroxyl radicals were in a concentration-dependent manner from 0.04–3mg mL^−1^. The IC_50_ value of NTWP-II was 0.82 mg mL^−1^. According to the previous study, polysaccharides with moderate molecular weights have been found to have strong antioxidant activity. In this study, NTWP-II showed strong hydroxyl radical scavenging activity. This might be attributed to moderate molecular weight and high contents of hydrogen. However, the relationships between antioxidant activity and physicochemical property or structural features of the polysaccharide have not been comprehensively understood.^28^

Superoxide anion radicals are weak oxidants and are thus not harmful to the body. However, its combination with hydroxyl molecules may damage DNA and other biomolecules. Scavenging effects of NTWP-II and V_C_ on superoxide radical were shown in Fig. 8c. For these two samples, scavenging activities of NTWP-II and V_C_ followed a dose-dependent manner at all tested concentrations. The scavenging effects significantly increased with increasing concentration from 0.04 mg mL^−1^ to 3.0 mg mL^−1^ and IC_50_ values for scavenging superoxide radical was 0.35 mg mL^−1^. The results indicated that NTWP-II has a noticeable superoxide radical scavenging activity.

Chelation is an important biological process, as iron is an essential metallic element for respiration, oxygen transport, and the activity of many enzymes for metabolism. Among the transition metals, Fe^2+^ is known as the most powerful pro-oxidant due to its high reactivity, which accelerates the reactions of oxidation through the Fenton reaction.^29^ The ferrous ion chelating activity of NTWP-II at different concentrations was shown in Fig. 8d and compared with EDTA in the equivalent concentration as a positive control. The metal chelating ability was recognized as a correlative activity to antioxidant. From Fig. 8d, we knew the chelating activity of NTWP-II exhibited a much weaker metal chelating ability. The chelating rate for NTWP-II was only 57.3% even at 5.0 mg mL^−1^, while that of EDTA was 60.1% at 0.6 mg mL^−1^. Metal chelation activity reduces the concentrations of the transition metals, thereby catalysing lipid peroxidation and thus reducing oxidative reactions. The reducing power results revealed that NTWP-II can act as electron donor compound, react with free radicals and convert them to more stable products terminating, therefore, reactions of the radical chain.

## 4. Conclusion

In order to increase the extraction yield of polysaccharides form the fruit of *Nitraria tangutorum* Bobr. The optimal extraction conditions with hot water extraction were optimized by BBD. The optimum variables given by BBD were as follows: extraction time 7 h, extraction temperature 60 °C, liquid–solid ratio 15 : 1. Under these conditions, the experimental yield was obtained as 14.01 ± 0.11% (*n* = 3), which corresponded with the predicted value well. The purified homogeneous polysaccharides NTWP-II was successfully obtained by DEAE-cellulose column and Sephadex G-200 column chromatography. In addition, chemical analysis indicated that the weight average molar mass (*M*_w_), polydispersity (PD, *M*_w_/*M*_n_) and *z*-average radius of gyration (*R*_z_) were 2.29 × 10^5^, 1.32, 15.22 by SEC-LLS. Monosaccharide analysis revealed that NTWP-II was composed of rhamnose, arabinose, mannose, glucose and galactose with the molar ratio of 1.14 : 2.5 : 3.00 : 2.69 : 5.28. Based on the analysis of monosaccharide composition, 1D and 2D NMR, the backbone structure of NTWP-II consisted mainly of (1→3)-β-galactose. Besides, and linked with α-mannose, α-arabinose and β-galactose by the (1→3) glycosidic linkage and (→1)-α-glucose of the side chain was obtained. NTWP-II exhibited positive radical scavenging activities against DPPH radical, superoxide anion and hydroxyl radicals, and metal chelating ability *in vitro*. Thus, NTWP-II could be explored as a natural antioxidant food ingredient. These might further provide theoretical basis for the widely application of NTWP-II in medicine and health care products.

## Conflicts of interest

There are no conflicts to declare.

## Supplementary Material
